# Association of financial hardship and survival in working-age patients following cancer diagnosis in Taiwan

**DOI:** 10.1093/oncolo/oyaf140

**Published:** 2025-06-17

**Authors:** Yi-Yun Claire Ciou, Li-Nien Chien

**Affiliations:** Institute of Health and Welfare Policy, College of Medicine, National Yang Ming Chiao Tung University, Taipei 11221, Taiwan; Institute of Health and Welfare Policy, College of Medicine, National Yang Ming Chiao Tung University, Taipei 11221, Taiwan

**Keywords:** financial hardship, financial toxicity, cancer, survival

## Abstract

**Background:**

Extreme income or asset loss as severe form of financial hardship (FH) has been linked to worse survival outcomes in cancer patients. This study aimed to assess the incidence, risk factors, and impact of severe financial hardship (SFH) on survival among working-age cancer patients in Taiwan’s universal healthcare system, using an objective measure for SFH.

**Methods:**

This study analyzed linked national longitudinal data for patients aged 20-63 years diagnosed with cancer between 2007 and 2018. Severe financial hardship was defined as household net income falling below the poverty threshold post-diagnosis. Propensity score matching (1:4) was used to balance baseline characteristics between SFH and non-SFH groups. Cox proportional hazard models were used to estimate the hazard ratio (HR) of outcomes.

**Results:**

Among 400 229 working-age cancer patients, the incidence of SFH was 4.7 per 1000 person-years (95% confidence interval [CI], 4.6-4.9) over a mean follow-up of 5.7 ± 4.3 years. Severe financial hardship was associated with younger age, male sex, advanced stage, and intensive treatments. Patients with SFH within 1 year of diagnosis had significantly lower survival, with an adjusted HR of 1.64 (95% CI, 1.56–1.72) for all-cause mortality compared to those without SFH. Notably, early stage patients with SFH faced a higher relative mortality risk than advanced-stage patients.

**Conclusions:**

Severe financial hardship substantially increases mortality among cancer patients in Taiwan, highlighting gaps in financial protection. Addressing SFH through implementing targeted policies and enhancing support mechanisms is essential to improve survival outcomes and reduce disparities in cancer care.

**Implications for Practice**

The association between severe financial hardship and increased mortality highlights the importance of coordinated clinical and policy responses. Integrating standardized financial hardship assessments into routine oncology care, expanding financial navigation services, and adapting reimbursement frameworks to better address the full cost of cancer care are critical steps toward mitigating financial toxicity and improving survival outcomes within universal healthcare systems.

## Introduction

The rising costs of cancer care, particularly the costs of cancer medications, have contributed to widespread financial hardship (FH) among patients and their families.^1^ Financial hardship is a multidimensional construct that involves material, psychological, and behavioral challenges.^2^ Material FH includes direct financial strain, such as out-of-pocket costs, debt accumulation, and catastrophic health expenditures, which often lead to income loss, asset depletion, and even bankruptcy.^1,3^ Financial hardship has been closely linked to a decline in health-related quality of life,^2,4^ and severe forms such as bankruptcy have been linked to increased mortality risk.^2,4,5^ Working-age cancer patients are particularly vulnerable due to ongoing employment, financial, and family responsibilities.^6,7^ Employment interruption and income loss following a cancer diagnosis can lead to long-term economic instability and have been associated with higher mortality risk.^3,6-9^

Despite the increasing acknowledgment of FH as a component of cancer care, several enduring barriers hinder its integration into clinical decision-making. These include limited consultation time, inadequate clinician training on treatment costs and insurance policies, discomfort with financial discussions, and the constraints of system-level reimbursement frameworks.^10,11^ Structural features of healthcare systems may contribute to financial burdens, potentially influencing treatment adherence and clinical outcomes. While most research on FH has originated from the United States,^12^ evidence from countries with universal health coverage such as Australia, Canada, and the United Kingdom remains limited, where FH may be underrecognized due to assumptions of financial risk protection.^13-16^

In Taiwan, oncologists face the added challenge of navigating a complex interplay between ethical obligations to provide optimal care and the realities of rigid, budget-driven policies. The National Health Insurance (NHI) system provides near-universal coverage while operates under a prospective global budget and an all-or-nothing reimbursement model that often delays access to new therapies.^17^ These structural characteristics, along with restrictive eligibility criteria and usage limitations, shift substantial financial responsibility onto patients.^18^ Despite these systemic challenges, the clinical consequences of FH, particularly its impact on survival, remain insufficiently characterized in Taiwan and represent an important but underexplored knowledge gap in countries with universal healthcare systems.

While previous studies have explored the prevalence and risk factors of FH through self-reported surveys, few have objectively examined whether economic hardship leads to poor outcomes in national cohorts. To address this gap, this study utilized a population-based cancer registry linked with beneficiary profiles to assess patient financial deterioration below the poverty line as a severe form of FH, referred to as severe financial hardship (SFH). Specifically, we aimed to (1) estimate the incidence and risk factors associated with SFH in working-age cancer patients within Taiwan’s universal healthcare system and (2) investigate the effect of SFH on cancer survival.

## Methods

### Definition of SFH

Severe financial hardship was defined as a transition from non-low-income to low-income household status, based on household financial resources falling below the government-defined poverty threshold.^17^ Under Taiwan’s NHI program, individuals are typically enrolled through their employer, labor union, or local district office. Unemployed individuals are insured as dependents of an employed spouse or direct relative. Certification of low-income household status, regulated by the Social Assistance Act, is determined by per capita income below the region-specific minimum living expense, limited household assets, and an evaluation of the financial capacity of family members to provide support.^19^ Therefore, enrollment in the low-income household program was used as a reliable proxy indicator for identifying SFH, reflecting a substantial decline in household financial status following a cancer diagnosis.

### Data source

This population-based cohort study used 3 comprehensive administrative datasets. The Taiwan National Health Insurance Research Database (NHIRD), managed by the National Health Insurance Administration, includes reimbursement claims for both outpatient and inpatient services. The NHIRD provides extensive data on beneficiaries, including monthly income for premium calculations, enrollment category, and catastrophic illness status, which exempts patients with severe illnesses from certain NHI payments and copayment.^20,21^The Taiwan Cancer Registry is a population-based system that tracks patients with cancer and requires all hospitals to submit detailed medical records including patients’ baseline characteristics, cancer diagnoses, and initial treatments.^22,23^ To verify patient survival and causes of death, this study used the National Death Registry, a highly accurate and complete population-based registry with algorithm to verify the cause of death.^24^ Furthermore, these datasets were linked using a unique encrypted identifier in accordance with the regulations of the Health and Welfare Data Science Center, Ministry of Health and Welfare, Taiwan. This study was approved by the Institutional Review Board of National Yang Ming Chiao Tung University (approval no. NYCU113137AE).

### Study cohort

**Figure 1.**
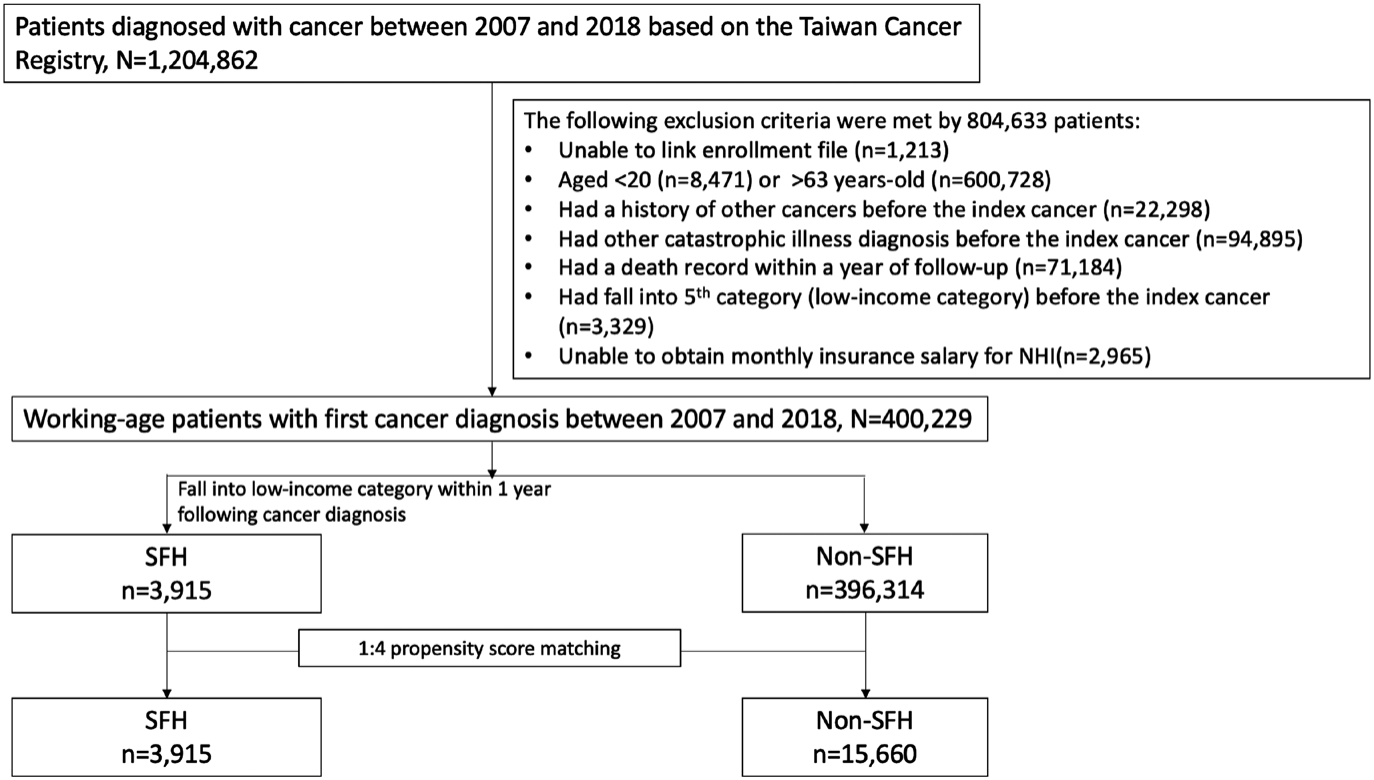
Patient selection process. SFH indicated transition to low-income household after cancer diagnosis. Abbreviations: NHI, National Health Insurance; SFH, severe financial hardship.

This study included patients who were first diagnosed with cancer (index cancer) between 2007 and 2018. The exclusion criteria were as follows: (1) inability to link enrollment data; (2) those younger than 20 or older than 63 years; (3) individuals with a history of any malignancy prior to the index cancer; (4) patients with a prior diagnosis of another catastrophic illness before the index cancer; (5) patients with a death record within 1 year following cancer diagnosis; (6) patients who had enrolled in the fifth category (low-income household) before cancer diagnosis; and (7) those without available monthly insurance salary data (Figure 1).

The upper age limit of 63 years was selected based on the planned retirement age reported in the annual workforce survey in order to minimize potential confounding related to changes in employment status. Additionally, to exclude patients with preexisting severe financial difficulties, individuals classified as low-income households before the index cancer diagnosis were also excluded. Patients who died within 1 year of their cancer diagnosis were also excluded, as early mortality is more likely to be attributable to disease severity than to financial factors.

We used 1:4 propensity score matching (PSM) to select a comparable cohort, balancing the distributions of the observed baseline characteristics between patients with SFH (defined as those classified as low-income household within a year after the index cancer) and non-SFH patients. Matched variables included age at diagnosis, sex, stage at diagnosis, monthly income at diagnosis, year of diagnosis, types of cancer treatment (surgery, systemic therapy, and radiation therapy), and the modified Charlson comorbidity index (CCI), which was calculated based on healthcare claims from the year preceding the index cancer diagnosis.

### Exposure and outcome definition

First, we examined the incidence and risk factors associated with SFH among working-age cancer patients as previously described.

The second outcome of interest was all-cause survival and cancer-related survival, defined as the interval from the date of the index cancer diagnosis to the date of death. All patients were followed up until the occurrence of outcomes or December 31, 2022.

### Study variables

Data on age, sex, cancer site, cancer stage, year of diagnosis, and the number of cancer treatments were obtained from the Taiwan Cancer Registry. To control for baseline socioeconomic status (SES), patients were classified into low, middle, and high SES groups based on tertiles of their individual monthly wage used for insurance premium calculation prior to the index cancer diagnosis. In addition, the severity of comorbidities was assessed using the modified CCI, excluding cancer-related diagnoses.

### Statistical analysis

We calculated the cumulative incidence of transitioning to the low-income category (defined as an SFH event) following the initial cancer diagnosis. The observational period was from the date of cancer diagnosis to the date of death or end of the study period, whichever occurred first. Cox proportional hazards analysis was conducted to identify the risk factors associated with SFH among working-age patients with their first cancer diagnosis.

Kaplan–Meier survival curves and log-rank tests were used to compare the probability of overall survival and cancer-specific survival in the SFH group who fell into low-income within 1 year of diagnosis and the non-SFH groups, respectively. Conditional Cox regression analysis was used to estimate the hazard ratios (HRs) of SFH for the mortality risk. The study endpoint was the date of death or the end of the observational period.

Baseline characteristics of patients in the SFH group were compared with those in the non-SFH group using the absolute standardized mean difference (ASMD), where an ASMD of less than 0.1 indicated negligible imbalance between groups.

A preplanned subgroup analysis was conducted to evaluate the outcomes of patients with and without SFH, including sex, age group, cancer stage, SES, and cancer site, as these factors are well documented in the literature to be associated with SFH. All statistical analyses were performed using SAS/STAT 9.4 (SAS Institute Inc., Cary, NC, USA) and STATA (version 16.0); StataCorp, College Station, TX, USA, with statistical significance set at *P* < .05.

## Results

### Characteristics of study participants

Patients newly diagnosed with cancer (*n* = 1 204 862) were identified from the Taiwan Cancer Registry between 2007 and 2018. Of these, 804 633 were excluded due to ineligibility, leaving 400 229 working-age patients with an initial cancer diagnosis included in the study (Figure 1). The mean follow-up duration was 5.7 years (standard deviation [SD] = 4.3). The analytic cohort had a mean age of 50.2 years (SD = 8.9), with 55.6% identifying as female. Most patients were diagnosed at an early stage (44.9%) and received one or more types of treatment (80.4%). Additionally, over 60% of patients had a modified CCI score of 0 (Table 1).

**Table 1.** Demographic and cancer-related factors of working-age patients with cancer by financial status within 1 year of cancer diagnosis before or after PSM.

					Before PSM^b^			After PSM^b^		
Variable	All (*n* = 400 229)		SFH^a^ (*n* = 3915)		Non-SFH (*n* = 396 314)		ASMD^c^	Non-SFH (*n* = 15 660)		ASMD^c^
	*n*	%	*n*	(%)	*n*	(%)		*n*	(%)	
Age at diagnosis [mean, SD]			[48.0, 7.7]		[50.2, 8.92]		0.257	[48.1, 8.5]		0.011
Age Group										
20-44	97 002	(24.2)	1304	(33.3)	95 698	(24.1)	0.203	5020	(32.1)	0.027
45-54	150 205	(37.5)	1737	(44.4)	148 468	(37.5)	0.141	7051	(45.0)	0.013
55-63	153 022	(38.2)	874	(22.3)	152 148	(38.4)	0.355	3589	(22.9)	0.014
Sex										
Male	177 835	(44.4)	2824	(72.1)	175 011	(44.2)	0.591	11 375	(72.6)	0.011
Female	222 394	(55.6)	1091	(27.9)	221 303	(55.8)	0.591	4285	(27.4)	0.011
SES^d^										
High	132 646	(33.1)	1834	(46.8)	130 812	(33.0)	0.285	7314	(46.7)	0.003
Medium	168 486	(42.1)	1278	(32.6)	167 208	(42.2)	0.198	5198	(33.2)	0.012
Low	99 097	(24.8)	803	(20.5)	98 294	(24.8)	0.103	3148	(20.1)	0.010
Cancer site										
Breast	88 851	(22.2)	432	(11.0)	88 419	(22.3)	0.306	1742	(11.1)	0.003
Head and neck	55 496	(13.9)	1364	(34.8)	54 132	(13.7)	0.51	5642	(36.0)	0.025
Colorectal	50 558	(12.6)	439	(11.2)	50 119	(12.6)	0.044	1758	(11.2)	<0.001
Female reproduction	36 618	(9.1)	176	(4.5)	36 442	(9.2)	0.187	681	(4.3)	0.007
Respiratory and chest	35 662	(8.9)	394	(10.1)	35 268	(8.9)	0.04	1548	(9.9)	0.006
Liver, gall bladder, Pancreas	31 545	(7.9)	301	(7.7)	31 244	(7.9)	0.007	1184	(7.6)	0.005
Endocrine	26 491	(6.6)	61	(1.6)	26 430	(6.7)	0.259	241	(1.5)	0.002
Other digestion system	18 792	(4.7)	334	(8.5)	18 458	(4.7)	0.157	1282	(8.2)	0.012
Leukaemia/lymphoma	16 814	(4.2)	144	(3.7)	16 670	(4.2)	0.027	564	(3.6)	0.004
Urinary system	15 335	(3.8)	76	(1.9)	15 259	(3.9)	0.114	264	(1.7)	0.019
Bone, joint, connective tissue, skin	10 483	(2.6)	72	(1.8)	10 411	(2.6)	0.053	264	(1.7)	0.012
Male reproduction	8431	(2.1)	38	(1.0)	8393	(2.1)	0.093	147	(0.9)	0.003
All others	5153	(1.3)	84	(2.1)	5069	(1.3)	0.067	343	(2.2)	0.003
AJCC Stage										
0	10 851	(2.7)	28	(0.7)	10 823	(2.7)	0.155	98	(0.6)	0.011
I	101 185	(25.3)	425	(10.9)	100 760	(25.4)	0.385	1672	(10.7)	0.006
II	67 629	(16.9)	562	(14.4)	67 067	(16.9)	0.071	2192	(14.0)	0.010
III	54 114	(13.5)	720	(18.4)	53 394	(13.5)	0.135	2865	(18.3)	0.002
IV	50 117	(12.5)	1154	(29.5)	48 963	(12.4)	0.431	4641	(29.6)	0.003
Unidentified^e^	116 333	(29.1)	1026	(26.2)	115 307	(29.1)	0.065	4192	(26.8)	0.013
Types of cancer treatment^f^										
0	77 666	(19.4)	790	(20.2)	76 876	(19.4)	0.020	3248	(20.7)	0.014
1	149 018	(37.2)	1108	(28.3)	147 910	(37.3)	0.193	4380	(28.0)	0.007
2	112 574	(28.1)	1258	(32.1)	111 316	(28.1)	0.088	5086	(32.5)	0.007
3	60 971	(15.2)	759	(19.4)	60 212	(15.2)	0.111	2946	(18.8)	0.015
Modified CCI										
0	264 850	(66.2)	2572	(65.7)	262 278	(66.2)	0.010	10 578	(67.5)	0.039
1-2	115 338	(28.8)	1092	(27.9)	114 246	(28.8)	0.021	4238	(27.1)	0.019
3	20 041	(5.0)	251	(6.4)	19 790	(5.0)	0.061	844	(5.4)	0.043
Year of diagnosis										
2007	26 788	(6.7)	258	(6.6)	26 530	(6.7)	0.004	1068	(6.8)	0.009
2008	28 178	(7.0)	284	(7.3)	27 894	(7.0)	0.008	1171	(7.5)	0.009
2009	30 192	(7.5)	339	(8.7)	29 853	(7.5)	0.041	1414	(9.0)	0.013
2010	32 049	(8.0)	336	(8.6)	31 713	(8.0)	0.021	1332	(8.5)	0.003
2011	32 788	(8.2)	440	(11.2)	32 348	(8.2)	0.104	1771	(11.3)	0.002
2012	34 125	(8.5)	380	(9.7)	33 745	(8.5)	0.041	1507	(9.6)	0.003
2013	34 316	(8.6)	375	(9.6)	33 941	(8.6)	0.035	1495	(9.5)	0.001
2014	35 222	(8.8)	312	(8.0)	34 910	(8.8)	0.030	1209	(7.7)	0.009
2015	35 967	(9.0)	330	(8.4)	35 637	(9.0)	0.020	1277	(8.2)	0.010
2016	35 452	(8.9)	301	(7.7)	35 151	(8.9)	0.043	1163	(7.4)	0.010
2017	37 412	(9.3)	266	(6.8)	37 146	(9.4)	0.095	1069	(6.8)	0.001
2018	37 740	(9.4)	294	(7.5)	37 446	(9.4)	0.07	1184	(7.6)	0.002

^a^SFH: transition to low-income household after cancer diagnosis. Abbreviations: AJCC, American Joint Committee on Cancer; ASMD, absolute standardized mean difference; CCI, Charlson Comorbidity Index; PSM, propensity score matching; SD, standard deviation; SES, socioeconomic status; SFH, severe financial hardships.

^b^Matched variables included age at diagnosis, sex, stage at diagnosis, socioeconomic status, year of diagnosis, type of cancer treatment and the modified CCI, year of cancer diagnosis.

^c^ASMD < 0.1 denotes no difference between the 2 groups.

^d^Patient monthly income was used to represent socioeconomic status.

^e^Unidentified AJCC stage: Some AJCC staging information was missing owing to variations in the availability of AJCC staging data across different periods.

^f^Types of Cancer treatment: 0 = no treatment; 1 = received surgery, radiotherapy, or systemic therapy; 2 = received 2 of the 3 treatment modalities (surgery, radiotherapy, or systemic therapy); and 3 = received all 3 types of treatment.

### Incidence and risk of SFH following cancer diagnosis

**Figure 2.**
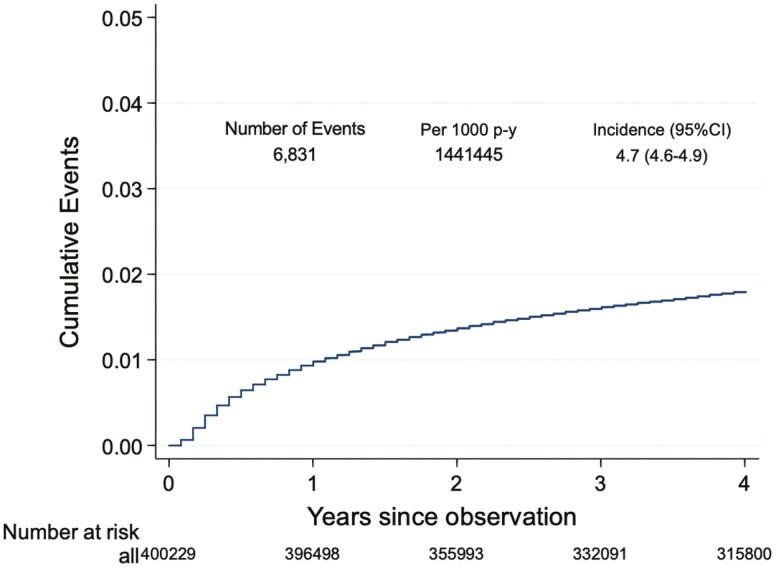
Cumulative incidence of SFH after first cancer diagnosis (*N* = 400 299). CI, confidence interval; p-y, person-years.

The incidence of SFH was 4.7 per 1000 person-years (95% confidence interval (CI), 4.6-4.9). Notably, 57.3% of the patients experienced SFH (3915 of 6831) within the first year following their cancer diagnosis (Figure 2).

Cox proportional hazard analysis identified key factors associated with SFH among working-age patients with cancer (Table 2). Younger age (20-44 years: adjusted hazard ratio (aHR) = 2.96, 95% CI = 2.77-3.17; 45-54 years: aHR = 2.02, 95% CI = 1.90-2.15) and male sex (aHR = 2.21, 95% CI = 2.05-2.38) were linked to a higher SFH risk compared to older patients and women. Conversely, medium (aHR = 0.53, 95% CI = 0.51-0.57) and low SES (aHR = 0.61, 95% CI = 0.57-0.65) were associated with lower risk compared to high SES. Advanced cancer stage, particularly Stage 4 (aHR = 3.55, 95% CI = 2.79-4.52), a modified CCI ≥ 3 (aHR = 1.66, 95% CI = 1.50-1.83), and receiving 3 types of treatments (aHR = 1.22, 95% CI = 1.12-1.34) were linked to higher SFH risk. Risk also varied by cancer site, with increased risk for digestive (aHR = 1.75, 95% CI = 1.57-1.96) and head and neck cancers (aHR = 1.71, 95% CI = 1.57-1.86), and lower risk for male reproductive (aHR = 0.43, 95% CI = 0.34-0.55) and endocrine cancers (aHR = 0.31, 95% CI = 0.25-0.38) compared to colorectal cancer. The risk of SFH declined over the diagnosis year, with the lowest in 2017 (aHR = 0.64, 95% CI = 0.57-0.73) and 2018 (aHR = 0.69, 95% CI = 0.61-0.79) compared to 2007.

**Table 2.** Cox regression analyses of the risk of SFH among working-age patients first diagnosed with cancer.

Variables	aHR^a^	(95% CI)	*P*-value^b^
Age group			
20-44	2.96	2.77-3.17	<.001
45-54	2.02	1.90-2.15	<.001
55-63	1.00	(Ref.)	
Sex			
Male	2.21	2.05-2.38	<.001
Female	1.00	(Ref.)	
SES			
High	1.00	(Ref.)	
Medium	0.53	0.51-0.57	<.001
Low	0.61	0.57-0.65	<.001
Cancer site			
Breast	0.99	0.88-1.11	.829
Head and neck	1.71	1.57-1.86	<.001
Colorectal	1.00	(Ref.)	
Female reproduction	1.18	1.03-1.36	.017
Respiratory and chest	1.27	1.15-1.42	<.001
Liver, gall bladder, pancreas	1.35	1.20-1.51	<.001
Endocrine	0.31	0.25-0.38	<.001
Other digestion system	1.75	1.57-1.96	<.001
Leukaemia/lymphoma	0.84	0.72-0.97	.017
Urinary system	0.80	0.67-0.95	.010
Bone, joint, connective tissue, skin	0.84	0.70-1.01	.063
Male reproduction	0.43	0.34-0.55	<.001
All others	1.66	1.39-1.99	<.001
AJCC Stage			
0	1.00	(Ref.)	
I	1.00	0.78-1.27	.975
II	1.72	1.35-2.19	<.001
III	2.37	1.86-3.01	<.001
IV	3.55	2.79-4.52	<.001
Unidentified^c^	2.10	1.65-2.67	<.001
Types of cancer treatment^d^			
0	1.00	(Ref.)	
1	0.90	0.83-0.96	.003
2	1.04	0.96-1.12	.384
3	1.22	1.12-1.34	<.001
Modified CCI			
0	1.00	(Ref.)	
1-2	1.06	1.00-1.12	.055
3	1.66	1.50-1.83	<.001
Year of diagnosis			
2007	1.00	(Ref.)	
2008	1.03	0.91-1.16	.631
2009	1.09	0.97-1.22	.167
2010	0.97	0.86-1.09	.615
2011	1.02	0.91-1.14	.749
2012	0.87	0.77-0.98	.019
2013	0.88	0.78-0.99	.030
2014	0.76	0.67-0.86	<.001
2015	0.79	0.70-0.90	<.001
2016	0.72	0.63-0.81	<.001
2017	0.64	0.57-0.73	<.001
2018	0.69	0.61-0.79	<.001

^a^Analysis adjusted for age group, sex, socioeconomic status, cancer site, AJCC stage, types of cancer treatment, modified CCI, year of diagnosis. Abbreviations: AJCC, American Joint Committee on Cancer; CCI, Charlson Comorbidity Index; Ref., reference group; aHR, adjusted hazard ratio; CI, confidence interval; SES, socioeconomic status.

^b^Two-sided test of statistical significance of differences in hazards from multivariable Cox proportion hazards model.

^c^Unidentified AJCC stage: Some AJCC staging information was unavailable owing to variations in the accessibility of AJCC staging data across different periods.

^d^Types of Cancer treatment: 0 = no treatment; 1 = received surgery, radiotherapy, or systemic therapy; 2 = received 2 of the 3 treatment modalities (surgery, radiotherapy, or systemic therapy); and 3 = received all 3 types of treatment.

### Impact of SFH on survival

The study cohort included 3,915 patients who experienced SFH during the first year post-diagnosis and 396,314 patients who did not. After applying PSM to account for differences in patient characteristics, both groups were found to be comparable in terms of the variables listed in Table 1.

**Figure 3.**
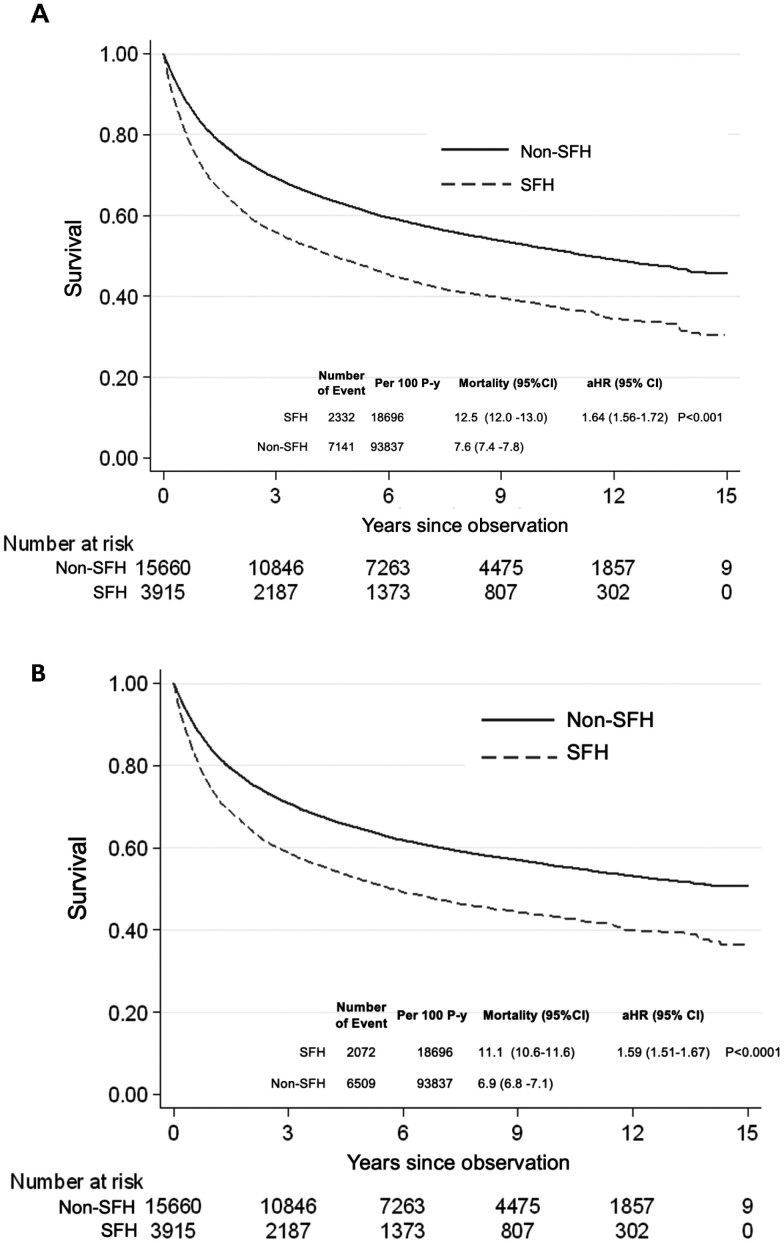
Overall survival and cancer-related survival among working-age patients with and without SFH. (A) Overall survival; (B) Cancer-related survival. The log-rank test was used to compare survival probabilities. Conditional Cox regression analysis was employed to estimate HRs of SFH for mortality risk. The analysis was adjusted for age group, sex, socioeconomic status, cancer site, AJCC stage, types of cancer treatment, modified CCI, and year of diagnosis. CI, confidence interval; p-y, person-years; aHR, adjusted hazard ratio; SFH, severe financial hardship.

Kaplan–Meier survival curves showed significantly lower overall survival (Figure 3A) and cancer-specific survival (Figure 3B) among patients with SFH compared to those without SFH. The all-cause mortality rate was 12.5 (95% CI, 12.0-13.0) per 100 person-years in the SFH group versus 7.6 (95% CI, 7.4-7.8) per 100 person-years in the non-SFH group. Similarly, cancer-related mortality rates were 11.1 (95% CI, 10.6-11.6) and 6.9 (95% CI, 6.8-7.1) per 100 person-years, respectively. Cox proportional hazards regression analysis revealed that the aHR for all-cause mortality was 1.64 (95% CI, 1.56-1.72) and for cancer-related mortality was 1.59 (95% CI, 1.51-1.67), indicating a significantly elevated risk of mortality among patients with SFH.

### Subgroup analysis

**Figure 4.**
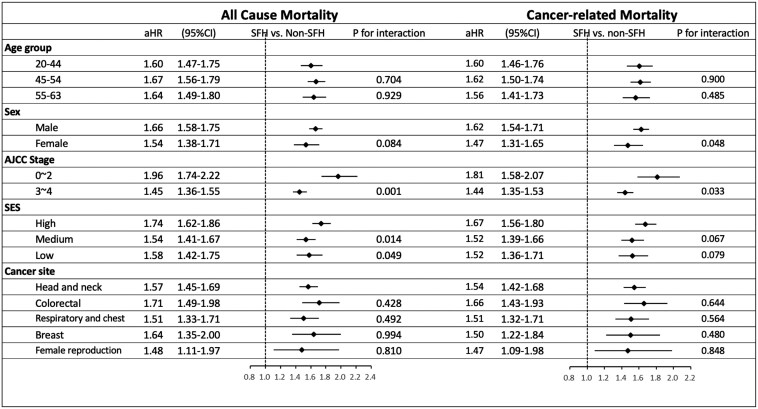
Adjusted HR of all-cause and cancer-related mortality in different subgroups of working-age patients with cancer with or without SFH. aHR, adjusted hazard ratio; CI, confidence interval; SES, socioeconomic status; SFH, severe financial hardship.

The analysis consistently showed that patients with SFH had higher risk of all-cause mortality than non-SFH patients, with aHRs ranging from 1.45 to 1.96, and similar results were observed for cancer-related mortality. The excess all-cause mortality risk associated with SFH was more pronounced in early stage cancer patients (aHR = 1.96, 95% CI = 1.74-2.22) than in those with advanced-stage disease (aHR = 1.45, 95% CI = 1.35-1.55), with a statistically significant interaction (*P* = .001). Notably, no significant interaction effects were observed for sex, specific cancer sites, or socioeconomic status on all-cause or cancer-related mortality (Figure 4).

## Discussion

In this large, nationwide cohort study spanning a decade, we found that patients with cancer in Taiwan who experienced SFH had a significantly higher mortality risk than those without SFH, based on a PSM-matched analysis. Our findings offer a novel alternative to studies that investigate the association between mortality and bankruptcy among patients with cancer,^5,25^ using the transition to low-income household status as a robust measure to indicate overall financial deterioration. Although relatively rare, this measure represents a severe form of FH that shares similarities with bankruptcy. As the out-of-pocket burden associated with cancer treatment continues to rise, our study contributes to growing evidence on the negative impact of FH on health outcomes.^12^ These findings emphasize the need for policies and interventions in oncology and survivorship care to alleviate SFH.

In our study of working-age patients with cancer in Taiwan, the incidence of SFH was relatively low, at 4.7 per 1000 person-years (95% CI, 4.6-4.9), measured by household income and assets falling below the poverty line. In contrast, in Washington State, only 3.2% of working-age cancer patients filed for bankruptcy after diagnosis.^26^ Surveys revealed significant disparities in FH between working-age and older cancer survivors, with working-age patients facing higher rates of FH (29.6%) than older patients (11.0%),^7^ likely due to employment disruptions such as reduced work hours, extended leave, or early retirement among younger patients,^27^ who also face additional burdens such as family and child-related expenses.^28^

Most risk factors identified in our study aligned with those in previous studies,^29-31^ although the higher risk among male patients and those with higher initial wages was a notable discrepancy. A possible explanation lies in the design of Taiwan’s NHI system, where monthly premiums are primarily based on an individual's regular earnings and closely tied to employment status. This method does not account for broader household-level financial conditions or obligations. In Taiwan, higher-income individuals tend to serve as the primary earners in their households, are more frequently male, and predominantly rely on wage-based income rather than investment returns or other passive income sources.^32^ Consequently, job loss due to illness may result in a more substantial and immediate decline in household income. A similar finding has been observed in the Netherlands, where income loss following major health shocks may be more pronounced in high-income groups, likely due to their stronger labor market attachment and higher pre-illness earnings.^33^ Moreover, studies from Taiwan have indicated that high-income individuals tend to incur higher out-of-pocket medical expenses, which may further exacerbate financial burdens during cancer treatment.^34-36^ Another key finding was the decline in SFH risk over the diagnosis year, with the lowest risk in 2017 compared with that in 2007. This trend may be related to the growing prevalence of private health insurance, which supplements public healthcare coverage. According to the Taiwan Insurance Institute, the average number of health insurance plans per person increased from 2.5 in 2007 to 3.6 in 2017, suggesting that private insurance may help protect cancer patients from severe financial deterioration.

We found that the risk of mortality for patients experiencing SFH was 1.6 times higher than that for those without SFH, consistent with studies in the United States where bankruptcy filings were associated with a 1.67 to 1.79 times higher mortality risk.^5,25^ Other studies based on self-reported financial difficulties showed a 17%-75% increase in mortality risk.^7,37,38^ Despite Taiwan’s universal healthcare and social support measures such as exemptions from NHI premiums, copayment, and subsidies for low-income households, cancer patients may still encounter substantial out-of-pocket costs. A survey of 3 hospitals in Taiwan found that 21.2% of patients had high medical expenses, which hindered treatment access,^39^ potentially leading to non-adherence to treatment, missed appointments, and poorer survival outcomes.

Severe financial hardship was associated with higher all-cause mortality across all subgroups, with a particularly strong and unexpected effect in early stage disease, suggesting that financial barriers may adversely affect outcomes even in curative-intent settings. The results were internally consistent (Table S1). One possible explanation is that FH may limit access to essential postsurgical adjuvant therapies, which are critical for recurrence prevention, require prolonged treatment, and were not fully reimbursed by Taiwan’s NHI during the study period. For example, patients receiving adjuvant therapy for early stage breast cancer could face out-of-pocket costs equivalent to up to 3 times the median annual per capita disposable income,^18^ whereas targeted therapies for advanced-stage disease were reimbursed. Cost-related barriers, such as insurance gaps and limited access to medications, have been shown to impair adherence among early stage patients,^40-42^ and poor adherence is associated with reduced 5-year survival and increased mortality risk (HRs ranging from 1.26 to 2.69).^43-45^

A key strength of this study is the use of a large national cohort linking 10 years of data from the NHIRD, Cancer Registry, and Death Registry, enabling objective tracking of financial status and mortality. To reduce bias, we defined SFH as significant financial deterioration after cancer diagnosis and excluded patients with preexisting low-income certification or catastrophic illnesses. To address survivorship bias, where sicker patients may die before receiving low-income certification, we excluded those who died within 1 year of diagnosis and used PSM to balance the cancer stage between the SFH and non-SFH groups. Survival outcomes were measured accurately using the validated National Death Registry. Nevertheless, this study had some limitations. While PSM balances observable characteristics, unmeasured confounders such as smoking or excessive alcohol use may still affect survival outcomes. In addition, lacking direct data on prior financial status and private health insurance coverage, we used surrogate indicators such as age, sex, and monthly salary, which may not fully capture the complexity of financial circumstances before diagnosis.

### Policy implementation

The observed association between FH and increased mortality among working-age cancer patients within a universal health system suggests the importance of both policy and clinical interventions. In Taiwan, recent policy initiatives have aimed to strengthen budget planning and enhance funding flexibility for high-cost oncology treatments. These include the implementation of a horizon scanning mechanism and proposals to establish a cancer-specific drug fund outside the NHI framework.^46^ Resource optimization has also been promoted through pilot programs encouraging the use of biosimilars.^47^ In addition, regulatory measures such as the introduction of parallel review processes and adjustments to reimbursement scheduling have been designed to reduce delays in access to new therapies.^46^

Equally important is the need to improve clinical recognition and management of FH. Oncology centers should be encouraged to adopt financial assessments as part of regular practice, facilitate cost-informed shared decision-making, and establish patient navigation systems that connect individuals to financial assistance and support services. Aligning policy and clinical efforts may help reduce financial burden and support equitable access to cancer care within resource-constrained systems.

## Conclusion

Severe financial hardship significantly increases the mortality risk among working-age cancer patients in Taiwan, despite universal healthcare coverage. These findings highlight critical gaps in financial support, indicating the need for integrated financial assessments, tailored assistance, and expanded policies to address the full costs of cancer care. Addressing FH is essential to improve patient outcomes and reduce health disparities.

## Supplementary Material

oyaf140_suppl_Supplementary_Tables_1

## Data Availability

The data underlying this study were provided by Health and Welfare Data Science Center (HWDC), Ministry of Health and Welfare in Taiwan. Due to the legal restrictions imposed by the government of Taiwan in relation to the Personal Information Protection Act, data cannot be made publicly available. Requests for data can be sent as formal proposals to the HWDC with IRB approval for research purposes only. In addition, these data can only be accessed and analyzed in an independent operating area of the HWDC. Only statistical results were obtained for the operating area. Therefore, the original data cannot be publicly shared owing to legal restrictions.
