# Supplementary material for: Association of financial hardship and survival in working-age patients following cancer diagnosis in Taiwan
Source: Oncologist. 2025 Jun 17;30(6):oyaf140. doi: 10.1093/oncolo/oyaf140 (PMC12200238; doi:10.1093/oncolo/oyaf140)
Supplement: oyaf140_suppl_Supplementary_Tables_1 [file oyaf140_suppl_supplementary_tables_1.docx]

**Supplementary Table 1: All-cause Mortality Rates per 1000 Person-Years Among Working-Age Cancer Patients, Stratified by Cancer Stage and SFH Status**

| **Variable** | | **All** | | **SFH** | | **Non-SFH** | |
| --- | --- | --- | --- | --- | --- | --- | --- |
| AJCC stage | | Mortality rate^*^ | (95% CI) | Mortality rate^*^ | (95% CI) | Mortality rate^*^ | (95% CI) |
|  | 0~2 | 3.64 | (3.45-3.84) | 5.74 | (5.19-6.35) | 3.17 | (2.98-3.38) |
|  | 3~4 | 13.25 | (12.91-13.6) | 18.27 | (17.3-19.3) | 12.26 | (11.9-12.63) |
|  | All | 8.83 | (8.62- 9.04) | 12.22 | (11.65-12.8) | 8.12 | (7.91- 8.34) |

*Events per 1,000 person-years
